# Haplotype association analysis of combining unrelated case-control and triads with consideration of population stratification

**DOI:** 10.3389/fgene.2014.00103

**Published:** 2014-04-29

**Authors:** Shu-Hui Wen, Miao-Yu Tsai

**Affiliations:** ^1^Department of Public Health, College of Medicine, Tzu-Chi UniversityHualien, Taiwan; ^2^Institute of Statistics and Information Science, National Changhua University of EducationChang-Hua, Taiwan

**Keywords:** case-parent trios, combined association analysis, haplotype weighted-count, haplotype specific association test, population stratification

## Abstract

Combining data when data are collected under different study designs, such as family trios and unrelated case-control samples, gains more power and is cost-effective than analyzing each data separately. However, a potential concern is population stratification (PS) among unrelated case-control samples and analyses integrating data should address this confounding effect. In this paper, we develop a simpler method, haplotype generalized linear model (HGLM), that tests and estimates haplotype effects on disease risk and allows for modification against PS for combining data. We proposed to combine information across aggregations of haplotype weighted-counts estimated from population case-control data and trio data separately, and to perform subsequent GLM analysis. Furthermore, we present a framework of analysis of variance based on haplotype weighted-counts for detecting whether it is appropriate to combine two data sources, as well as the modified HGLM with clustering methods for addressing PS. We evaluate the statistical properties in terms of the accuracy, false positive rate (FPR) and empirical power using simulated data with regard to various disease risks, sample sizes, multi-SNP haplotypes and the presence of PS. Our simulation results indicate that HGLM performs comparably well with the likelihood-based haplotype association analysis, particularly when the haplotype effects are moderate, but may not perform well when dealing with lengthy haplotypes for small sample sizes. In the presence of PS, the modified HGLM remains valid and has satisfactory nominal level and small bias. Overall, HGLM appears to be successful in combining data and is simple to implement in standard statistical software.

## Introduction

Genetic association analysis has been used widely in searching for genes contributing to complex diseases, for example, several large-scale genetic studies collected genome data from both typically healthy and diseased individuals in genetic association tests (Hunter et al., 2007; Kibriya et al., 2009). One very common design is to collect unrelated population data such as case-control data as the power is larger than with traditional linkage studies in detecting mild effects (Risch and Merikangas, 1996). However, one drawback is spurious association resulting from population stratification (PS). Therefore, careful correction for PS is essential when analyzing case-control studies. To avoid spurious association, family-based design is an alternative method and using case-parent trios is an appealing strategy (Spielman et al., 1993; Cardon and Palmer, 2003). In other words, we can observe genotypes of diseased offspring and those of his/her two parents where the non-transmitted parental alleles are viewed as a pseudo-control sample. However, the recruitment of parents is often more difficult for late onset diseases and more expensive than recruiting unrelated controls. Such difficulty involved in data collection and/or cost-burden might result in power loss with regard to detecting associated markers.

A number of methods have been developed for combining samples consisting of unrelated case-control data and family data. A notable strength is the increase in power with regard to identifying associated markers (Glaser and Holmans, 2009) but the pooling data requires deliberation in statistical analysis. One strategy was to provide a weighted estimate of disease risks from separate analyses of population case-control and family trios. Kazeem and Farrall (2005) estimated the allelic odds ratio (OR) of disease association separately and provided a weighted logarithm of two separate ORs for inference. Chen and Lin (2008) proposed a weighted least square estimator for the logarithm of genotype relative risks in a logistic regression model with genotype covariates and constructed a Wald-type test employing joint information from combined data. From a statistical hypothesis testing perspective, Joo et al. (2007) presented weighted average test statistics composed of the transmission disequilibrium test (TDT) (Spielman et al., 1993) for trio data and trend test for case-control data. Recently, Stewart and Cerise (2013) proposed a combination test of a trend test and a Wald test without any assumptions about the genetic model. The other strategy was a likelihood-based approach dealing with genotype data combining trios and unrelated controls. The combined likelihood for pooling data is formulated by multiplying the likelihood contributions with regard to family data as well as unrelated controls. Nagelkerke et al. (2004) constructed a likelihood function of the genotype risk ratio based on a multiplicative model and utilized a traditional Poisson regression model to estimate the ratio. However, this requires restrictive model assumptions such as rare disease assumption and Hardy-Weinberg equilibrium. Epstein et al. (2005) further utilized the approach conditional on the parental genotypes to infer the risk ratio from the likelihood function. They found that the power is similar to that of Nagelkerke et al.'s approach with less restrictive assumptions; hence, a formal test for the adequacy of data combining was conducted. However, these approaches are limited to the analysis of one marker at a time and no other covariates. For analyzing multiple markers, a unified likelihood framework was developed to test for the disease-marker association (Li et al., 2006; Dudbridge, 2008). For more flexible modeling, Hsu et al. (2009) proposed a pseudo-likelihood approach that models the probability of genotypes conditional on the disease phenotype and covariates. Pfeiffer et al. (2008) proposed a random effect model incorporating genotype data of family trio and population case-control data. Wang et al. (2013) proposed a multi-marker VC-based association test using both family and unrelated data with allowing for the adjustment of non-genetic contributions to the familial similarity, as well as multiple covariates. These approaches appear to be far more attractive because of the flexibility by which covariates are known or suspected to affect risk incorporated into the analysis. Recently, some strategies were also developed to account for PS without homogeneous population assumption (Chung et al., 2010; Mirea et al., 2012).

Apart from that mentioned research-article approaches based on haplotypes, which refer to a set of alleles at different loci that are present together on the same chromosome, are proposed for combined analysis. For example, Guo et al. (2009) advocated a combined haplotype relative risk (CHRR) that is an allele-based association test. They claimed that the CHRR is more powerful than Epstein et al.'s strategy in the presence of linkage. Particularly, Dudbridge (2008) developed a likelihood-base approach for combined data on the basis of haplotype inference. Also, there is some theoretical evidence that haplotype-based tests would be more powerful. Because single marker linkage-disequilibrium (LD) based methods may not capture all of the available LD information, which is contained in multi-locus haplotypes (Akey et al., 2001; Schaid, 2005). General features related to combined methods are (1) utilizing various components of available population and family data, and (2) carrying out analyses using either per SNP at a time or haplotype-based data, (3) obtaining a single risk estimate from combined data or a weighted estimate based on distinct estimates from separate analyses, and (4) further testing for PS since validity of disease risk estimates for most of existing methods depends on PS absent (Infante-Rivard et al., 2009; Fardo et al., 2011). The first feature is limited to practical situations such as resources collected from unrelated case-control and family trio data or unrelated controls and case-parent trios. One of our goals is to present strategy on how and when unrelated case-control data and independent case-parent trios may be combined. For dealing with combined data and detecting for the presence of PS efficiently, we propose a haplotype generalized linear model (HGLM) based on haplotype weighted-counts from the combined data. Similar to two-stage approaches (Schaid et al., 2002; Zaykin et al., 2002; Sham et al., 2004; Purcell et al., 2007), we use estimated haplotype weighted-counts rather than haplotype probabilities as predictor variables, and then perform haplotype association analyses in a GLM framework. Except for homozygous and single heterozygous subjects, haplotypes are generally not observed but can be inferred statistically. The entries in predictor variables corresponding to haplotypes are no longer 0, 1, and 2, reflecting the phase uncertainty. Subject-specific haplotype distributions can be obtained from the conditional likelihood given the individual's genotype and the estimated haplotype frequencies (HFs). We propose to combine information across aggregations of haplotype weighted-counts estimated from population case-control data and family trio data separately, and to perform subsequent GLM analyses. The method we present here is similar to the method of Zaykin et al. (2002) which consists of a simpler expectation substitution method. As in a single marker situation, there are no phase ambiguities; HGLM using only population case-control data would reduced to the method of Zaykin et al. (2002) in haplotype-based data.

The proposed HGLM has some advantages. First, many available programs are capable of haplotype reconstruction (e.g., SNPHAP or PHASE or FAMHAP software); we have chosen the FAMHAP program in our implementation. Then it is quite simple to perform the haplotype analysis with standard statistical software. In contrast, inference based on the joint likelihood of both HFs and haplotype disease risks (e.g., Dudbridge, 2008) will be more complicated although programs available for this task such as Unphased (Dudbridge, 2008). The second advantage is the simple aggregations of haplotype weighted-counts from population case-control data and family trio data, and we prove that the estimates of haplotype effects based on the aggregations of haplotype data are accurate by our simulation results. In other words, we use the full information of “pooled sample” rather than composite likelihoods obtained from the joint likelihood of both haplotype probabilities and disease models for either data. We further develop a modified HGLM to deal with the confounding effect of PS through a clustering technique without complexity. Here, we propose the implementation of HGLM for combining two types of data and compare statistical properties with the existing method in Dudbridge (2008) in terms of bias, false positive rate (FPR) and power. The performance of the proposed HGLM is evaluated by a variety of simulation studies such as various haplotype disease risks, sample sizes, multiple markers and the presence of PS. As we shall see later, HGLM performs comparably well to the method in Dudbridge (2008) with regard to PS.

## Methods

### Haplotype generalized linear model (HGLM)

Suppose genetic data are collected from *n*_1_ case-parents trios and unrelated case-control samples (*n*_2_ cases and *n*_3_ controls) that are sampled from the same population, and each individual is genotyped at *q* biallelic SNPs while two alleles are denoted by 1 and 2. For a given triad, G_p_ = (G_f_, G_m_) and G_o_ are defined as the genotypes of the two parents and of the affected probands, respectively. G_u_ denotes the genotypes of unaffected controls and G_a_ refers to the genotypes of the affected cases for population case-control data. For estimating the association of specific haplotypes with disease phenotype, we propose HGLM with the combined haplotype weighted-count data as covariates which are estimated from family trios (i.e., G_p_ and G_o_) and population case-control genotype data (i.e., G_u_ and G_a_) separately. Suppose the size of groups including trios, population cases, and controls is *n* = *n*_1_ + *n*_2_ + *n*_3_. Let *y*_*ij*_ be the disease phenotype (1 = affected and 0 = unaffected) of the *j*th subject for the *i*th group (*i* = 1,…, *n*), where *j* = 1 and *j* = 2 represent an affected proband and a pseudo control for trio data, respectively, and *j* = 1 for population case-control data. Following the proposal in Falk and Rubenstein (1987), we use the affected offspring as a case individual and construct a single pseudo-control individual with phased genotypes consisting of the haplotypes not transmitted from the parents to the affected offspring. For *n*_1_ trios, the affected probands (*y*_*ij*_ = 1) are the cases with transmitted haplotypes and pseudo controls (*y*_*ij*_ = 0) are with non-transmitted haplotypes. Therefore, the total number of cases and controls is *N* = 2*n*_1_ +*n*_2_ +*n*_3_ for our analysis. The general model setting for the combined analysis is

$$\begin{array}{l} \text{logit}[P(y_{ij}\;=\;1|H_{ij})]=\alpha +{H^{\prime }}_{\text{ij}}\beta \\ y_{ij}\;=\;\{\begin{array}{l} 1\;if\;i\leq n_{1},\;j=1\;or\;n_{1}+1\leq i\leq n_{1}+n_{2},\;j=1\\ 0\;if\;i\leq n_{1},\;j=2\;or\;n_{1}+n_{2}+1\leq i\leq n,\;j=1 \end{array} \end{array}$$

where *α* is the intercept as the effect of a baseline haplotype and **β** = (*β*_1_, *β*_2_, …, *β*_*p*_)′ represents the *p* × 1 vector of the logarithm of ORs for specific haplotypes. The coding of **H**_*ij*_ = (*h*_1*ij*_, *h*_2*ij*_,…, *h*_*pij*_)′ denotes the *p* × 1 vector of weighted-counts for haplotypes of the *j*-th subject for the *i*-th group, and it contains a pooled set of haplotype weighted-counts obtained from trios (i.e., *i* ≤ *n*_1_) and population case-control data (i.e., *n*_1_ + 1 ≤ *i* ≤ *n*). Let *p* = 2^*q*^ −1 be the number of the distinct haplotypes observed in the combined data by excluding the most common haplotype *h*_0_ as the reference haplotype. The weighted-count can be defined as the number of haplotype occurrence (i.e., 0, 1, 2) multiplies by corresponding weight which means the estimated haplotype probability. For homozygous genotypes at *q* SNP sites or at most one heterozygous SNP, the weighted-count would be either 0 or 2 due to no haplotype ambiguity. However, the weight-count would range from 0 to 2 if the subject is heterozygous at more than one SNP. The case and control haplotypes are estimated by applying an expectation-maximization algorithm such as the Famhap program written by Becker and Knapp (2004). A wide variety of programs exists for haplotype reconstruction based on unphased genotype data. One could choose a preferred program to obtain **H**_*ij*_ = (*h*_1*ij*_, *h*_2*ij*_,…, *h*_*pij*_)′.

**Figure 1:**
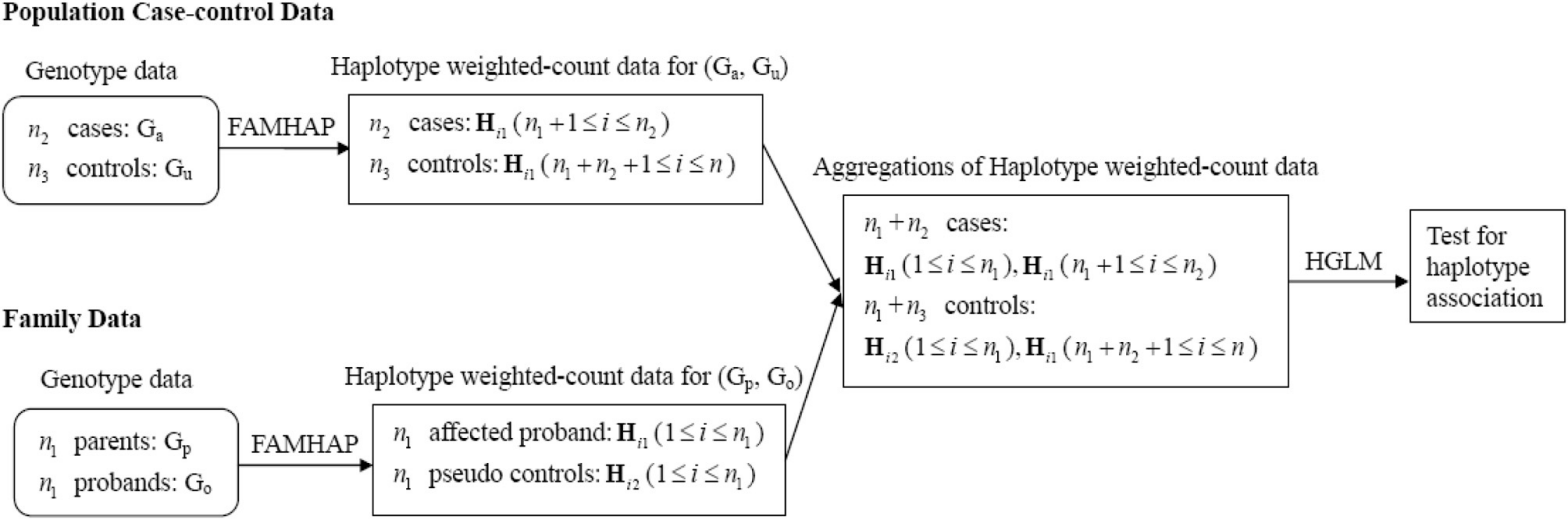
**Schematic diagram of HGLM for combining data of unrelated case-control samples and family trios**.

We perform a sequential analysis for fitting HGLM. The schematic diagram of HGLM for combining data of unrelated case-control samples and family trios is depicted in Figure 1. First, the family trios (i.e., G_p_ and G_o_) data are analyzed in the Famhap program to obtain the haplotype explanation files, as well as for the population case-control data (i.e., G_u_ and G_a_) as a single data set. These outputs from Famhap are reorganized as the haplotype weighted-count data, and these weighted-count data from population and family studies are combined in HGLM. For either case/pseudo control of family data or each case/control of population data, the sum of weighted-counts of all possible haplotypes should equal two. This would lead to obtain unidentifiable regression coefficients (i.e., disease risks). Hence, the most common haplotype *h*_0_ is removed in the fitted HGLM model allowing a regression coefficient *β*_*k*_ to be interpreted as the log(OR) of disease for the haplotype *h*_*k*_ relative to the haplotype *h*_0_, *k* = 1,…,*p*. Additionally, testing the effect of a specific haplotype on disease risk, called the haplotype-specific association test, is performed based on a Wald test or score test, and the confidence intervals of haplotype-specific ORs can be provided easily.

### Testing and accounting for population stratification

It is possible that the heterogeneity of haplotype effects may present between families and unrelated subjects that might be introduced if HFs (resulting from different minor allele frequencies) and disease probabilities differ among population case-control samples (that is PS), and therefore combining two data sources directly is inappropriate. In other words, if there is PS among population case-control samples or two data sources come from different populations, it would result in different means of haplotype weighted-counts for case-control samples as compared with family trios. Hence, we propose a multivariate analysis of variance (MANOVA) model as a tool for detecting the appropriateness of combining data. The MANOVA model is

$$H_{ijg}=\mu +\tau_{g}+\varepsilon_{ijg},g=1,2$$

where **H**_*ijg*_ = (*h*_1*ijg*_, *h*_2*ijg*_, …, *h*_*pijg*_)′ means the weighted count of the *j*th subject for the *i*th group from either family trios (if *g* = 1) or population case-control samples (if *g* = 2), ***τ***_*g*_ denotes the *p* × 1 vector of the effect for each data source (*g* = 1,2), and ***ε***_*ijg*_ is the random error term. The hypothesis is given by *H*_0_ : ***τ***_1_ = **τ**_2_ = 0 vs. *H*_1_ : at least one **τ**_*g*_ ≠ **0**. If the null hypothesis is rejected, it implies that the means of haplotype weighted-counts of family trios and population case-control data are significantly different, and therefore, combining two data sources directly is inappropriate. Subsequently, we can perform ANOVA for comparison of the specific HFs between family trios and population case-control data. The ANOVA model for the haplotype *h*_*k*_ is

$$h_{kijg}=\mu +\tau_{kg}+\varepsilon_{kijg},g=1,2$$

where *h*_*kijg*_ represents the weighted count of the *j*th subject within the *i*th group from either family trios (if *g* = 1) or population case-control samples (if *g* = 2) for the haplotype *h*_*k*_, *τ*_*kg*_ denotes the effect of each data source (*g* = 1,2) for the haplotype *h*_*k*_. Similarly, if the null hypothesis *H*_0_ : *τ*_*k*1_ = *τ*_*k*2_ = 0 is rejected, it indicates that the means of weighted-counts for the haplotype *h*_*k*_ obtained from the family trios and population case-control data are significantly different. It is worth noting that the proposed tests for PS cannot distinguish between the confounding effect by differences in HFs between family trios and unrelated case-control samples (i.e., PS) and true heterogeneity of haplotype effects. In either case, however, it would not be appropriate to combine the family data with unrelated case-control samples. Therefore, the proposed test will be a useful indication of whether to combine the data or not. The similar opinion has been elicited by Infante-Rivard et al. (2009).

**Figure 2:**
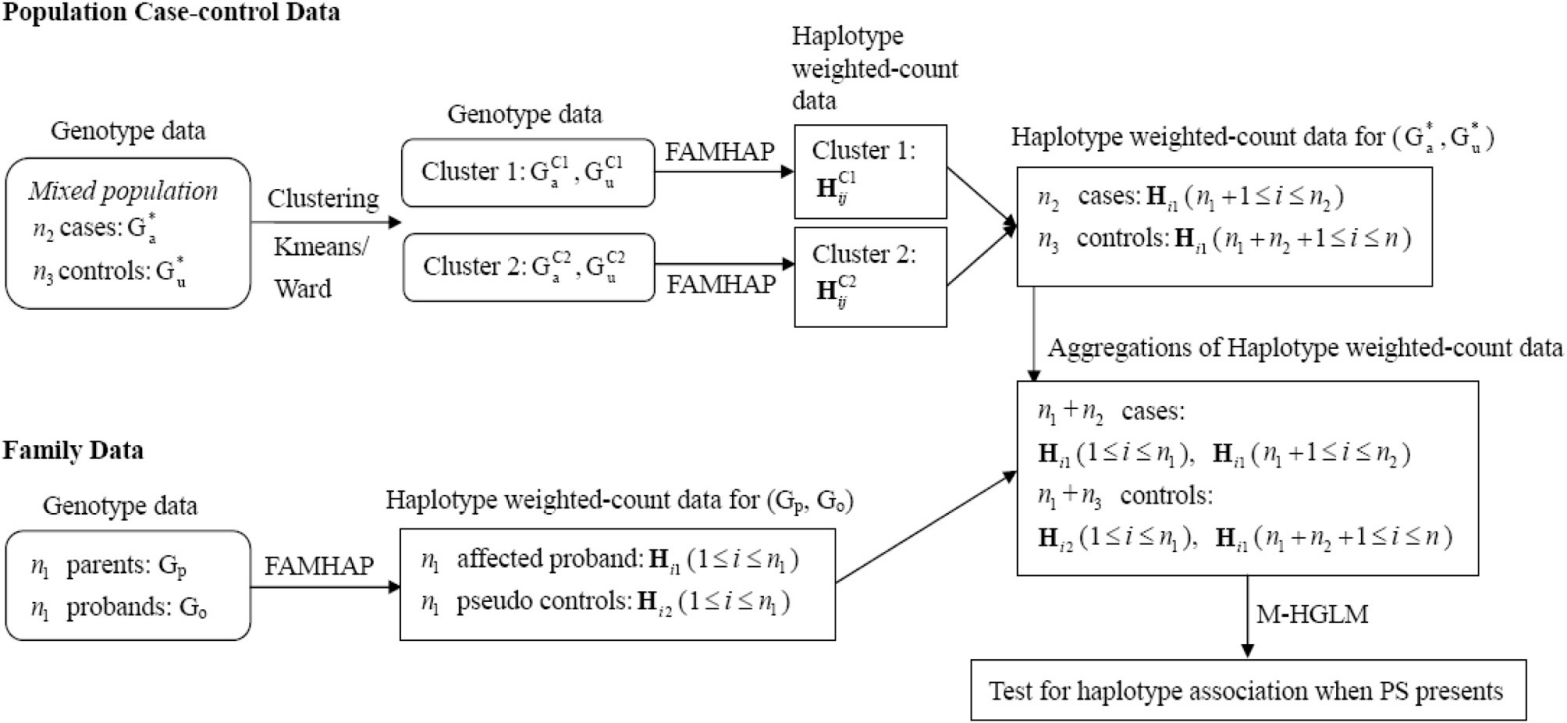
**Schematic diagram of M-HGLM for combining data of clustered case-control samples and family trios when PS presents. H**^C1^_*ij*_ and **H**^C2^_*ij*_ denote the haplotype weighted-count obtained from clusters 1 and 2, respectively, when performing clustering methods on mixed population case-control genotype data.

When there is PS, the estimate of disease risk would be biased and the FPR would be inflated. HGLM can be modified further for addressing PS by calculating haplotype weighted-counts based on homogeneous subgroups, such that measures of association are not confounded. To account for the bias of PS, we utilize clustering methods to group the mixed case-control samples into clusters based on genotype data (i.e., G^*^_u_ and G^*^_a_), and use the Famhap program to obtain estimates of haplotype weighted-count data from each cluster (i.e., homogeneous subgroups) separately. Since family trio data are robust against PS (Spielman et al., 1993; Cardon and Palmer, 2003), the haplotype weighted-count data estimated from mixed trio data are still valid within expectation. Thus, HGLM can be modified and implemented by combining haplotype weighted-count data estimated from family trios and clustered case-control samples. The schematic diagram of the modified HGLM (termed M-HGLM) for combining data of mixed case-control samples (when PS presents) and family trios is depicted in Figure 2. For clustering of case-control samples from admixed populations, we use a genotype data matrix (the entries are 0, 1, or 2 representing the number of copies of the derived allele) with one row for each of *n*_2_ cases and *n*_3_ unrelated controls, and one column for each of *q* bi-allelic loci. We normalize genotype data by column means and standard deviations. The distance matrix between all pairs of individuals is constructed based on Euclidean distance of multi-locus normalized genotype data. The normalized matrix is then used for clustering method including Ward's hierarchical clustering approach and a non-hierarchical clustering method, K-means algorithm. Ward's method starts out with *n*_2_ + *n*_3_ clusters of size 1 and continues until all the individuals are grouped into one cluster. Given a user specified number of subgroups, Ward's algorithm is performed to identify clusters where the error sum of squares of individuals from two joined clusters is minimized. As for K-means algorithm, the number of clusters (K) may be specified in advance. In this paper, Ward's clustering algorithm and K-means algorithm are implemented with the standard statistical software, R, using the function hclust() and kmeans(), respectively.

## Simulations

We performed simulations to assess the statistical properties and power of our proposed combined method HGLM across a range of degree of haplotype risks, sample sizes, multi-SNP haplotypes and strength of PS. Furthermore, we compared the power of HGLM with other approaches, UNPHASED (Dudbridge, 2008) and SCOUT (Epstein et al., 2005) that can also use both family and case-control data. SCOUT is restricted to use combined data based on a single marker test, while HGLM and UNPHASED (denoted as Unph) can use combined data on the basis of haplotype inference. We generated marker genotypes at 2 SNPs and multi-SNP as the genetic data in practice when performing the combined analysis.

First, haplotype sequence simulations were based on disease models including mild and moderate risks. A total of 20,000 haplotype sequences consisted of two biallelic markers were simulated with HFs (HFs) of 1-1, 1-2, 2-1, and 2-2 as 0.5, 0.1, 0.3, and 0.1, respectively. We generated case and control haplotype sequences from the logistic regression model with given haplotype risks ***β*** = (*β*_1_, *β*_2_, *β*_3_)′:

$$\begin{array}{l} \text{logit}[P(y_{ij}\;=\;1|H_{ij}]=\alpha +\beta_{1}I(h_{1ij}=1-2)\;+\;\beta_{2}I(h_{2ij}=2-1)\\ \;+\beta_{3}I(h_{3ij}=2-2) \end{array}$$

where *I*(·) denotes the indicator function and the parameters *β*_1_, *β*_2_, and *β*_3_ are the log odds ratios corresponding to the haplotype-specific risk of the 1-2, 2-1, and 2-2 haplotype vs. 1-1 haplotype. We set the values of haplotype odds ratios **β** = (log(1.207), (1.421), (1.525))′for a mild effect model, and **β** = (log(1), (2.067), (2.067))′ for a moderate effect model, respectively. We also simulated data where none of the haplotypes spanned by the markers have OR > 1 (i.e., all *β* = 0; denoted as null model) and assessed the FPR of the combined haplotype-specific association test. The intercept value of α was chosen to yield the disease rates of different disease models (i.e., null, mild, and moderate effect models) approximately at 5–7%. Next, individuals' genotypes in case and control groups were generated by randomly drawing a pair of haplotypes from the case haplotype sequences and from the control haplotype sequences, respectively. For family trio data, the disease outcome was further generated by a conditional logistic regression model (denoted as CLG) because each family was treated as a matched set:

$$\begin{array}{l} P(y_{i1}=1,y_{i2}=0|H_{ij})\\ \;=\frac{\begin{array}{ll} & \exp[\beta_{1}I(h_{1i1}=\;1-2)+\beta_{2}I(h_{2i1}=2-1)\\ & \;+\beta_{3}I(h_{3i1}=\;2-2)] \end{array}}{\begin{array}{ll} & \sum_{j\;=\;1}^{2}\exp[\beta_{1}I(h_{1ij}=1-2)+\beta_{2}I(h_{2ij}=2-1)\\ & \;+\beta_{3}I(h_{3ij}=2-2)] \end{array}} \end{array}$$

and *P*(*y*_*i*1_ = 0, *y*_*i*2_ = 1|**H**_*ij*_) = 1 − *P*(*y*_*i*1_ = 1, *y*_*i*2_ = 0|**H**_*ij*_), given by the haplotype weighted-count specified according to the same haplotype frequency and disease rate as population case-control data, as well as the same disease risk. In other words, we considered that the population case-control data and family trios were sampled from the same population (scenarios without PS). The diplotype data (haplotype pair) for affected children were generated first, followed by the parents' corresponding to random mating assumption. As for multi-SNP haplotype inference, we followed the simulation settings in Purcell et al. (2007) as shown in their Table 1 for haplotype-based association studies. A total of 100,000 haplotype sequences consisted of five biallelic markers and six haplotypes were simulated. The six HFs of 1-2-1-2-2, 1-1-1-1-1, 1-1-2-1-1, 1-2-2-2-2, 2-2-2-2-1, and 2-2-2-2-2 are 0.264, 0.169, 0.067, 0.050, 0.212, and 0.237, respectively. The reference haplotype is 1-2-1-2-2, and the values of haplotype odds ratios for the corresponding to the haplotype-specific risk of the other five haplotypes vs. the reference haplotype are set as ***β*** = (log(1.467), (3.811), (1.528), (1.309), (1.501))′. For all simulations, we set two sample sizes with equal and different numbers of samples from family trios and population case-control data (*n*_1_ = *n*_2_ = *n*_3_ = 100, and *n*_1_ = 500 and *n*_2_ = *n*_3_ = 1000) for the 2-locus mild and moderate effect models, and the multi-SNP model.

**Table 1:** **False positive rate under all haplotype odds ratios (HOR) equal to 1**.

**Null model**		**True HOR**	**Population data**			**Family data**			**Combined data**	
**Sample size**	**Haplotype**		**Estimate**	**P-HGLM**	**P-Unph**	**F-HGLM**	**F-Unph**	**F-CLG**	**C-HGLM**	**C-Unph**
	Power of the overall test			0.055	0.057	0.056	0.055	0.055	0.056	0.053
*n*_1_ = 100	1-2	1	FPR	0.044	0.047	0.050	0.049	0.047	0.043	0.036
*n*_2_ = *n*_3_ = 100			Bias	0.104	0.096	0.075	0.077	0.084	0.036	0.018
			MSE	0.311	0.300	0.195	0.201	0.228	0.089	0.062
	2-1	1	FPR	0.048	0.054	0.050	0.048	0.051	0.064	0.045
			Bias	0.047	0.045	0.028	0.026	0.030	0.019	0.009
			MSE	0.081	0.078	0.067	0.065	0.073	0.035	0.025
	2-2	1	FPR	0.045	0.043	0.050	0.045	0.043	0.050	0.041
			Bias	0.075	0.080	0.077	0.080	0.081	0.035	0.029
			MSE	0.224	0.235	0.183	0.184	0.192	0.078	0.057
	Power of the overall test			0.045	0.044	0.049	0.048	0.047	0.047	0.035
*n*_1_ = 500 *n*_2_ = *n*_3_ = 1000	1-2	1	FPR	0.043	0.046	0.034	0.035	0.032	0.042	0.037
			Bias	0.006	0.006	0.018	0.019	0.019	0.006	0.005
			MSE	0.017	0.017	0.025	0.026	0.026	0.010	0.008
	2-1	1	FPR	0.050	0.051	0.047	0.047	0.048	0.044	0.036
			Bias	0.001	0.001	0.008	0.008	0.008	0.001	0.001
			MSE	0.006	0.006	0.011	0.011	0.012	0.004	0.003
	2-2	1	FPR	0.050	0.048	0.043	0.043	0.041	0.049	0.035
			Bias	−0.001	−0.001	0.009	0.009	0.010	−0.002	−0.003
			MSE	0.014	0.014	0.026	0.027	0.027	0.009	0.007

The reference haplotype is 1-1.

To determine the statistical properties between our proposed method and test based on separate data, we conducted three ways of incorporating data for each data set: (1) population case-control data only, (2) family trios only or, (3) a combination of population case-control data and family trios. For family trios only, we adopted the CLG as a compared approach by using the transmitted and non-transmitted haplotypes matched within a family. We examined the performance of the proposed method in terms of bias, mean square error (MSE) and coverage rate of 95% confidence interval (CI) of estimates for haplotype odds ratio (HOR) over 1000 replicates. Furthermore, the empirical FPR and power were used to evaluate the performance of our haplotype-specific association test. Under the null hypothesis of no haplotype association (i.e., HOR = 1), the fraction of times that the *p*-values of haplotype-specific association tests less than 0.05 is the empirical FPR. The power is defined as the proportion of *p*-values of haplotype-specific association tests less than 0.05 while the haplotype-specific association exists. We implemented the simulations and computations using R 2.15.1 programming language and the code can be obtained from the authors.

## Results

### Null model: FPR, BIAS, and MSE

Table 1 shows the FPR, bias and MSE for our proposed HGLM, CLG, and Unph methods with two sample sizes at *n*_1_ = *n*_2_ = *n*_3_ = 100, and *n*_1_ = 500 and *n*_2_ = *n*_3_ = 1000. For Unph of Dudbridge (2008), the FPR can only be determined from the analysis output as the proportion of 1 lying outside of the 95% CI for HOR = 1 over 1000 replications. The results indicate that the FPRs under the null hypothesis (HOR = 1) are generally close to the nominal level at 0.05 for either two types of data analyzed separately or combined data for all methods examined. For the combined data analysis, HGLM yields smaller bias and MSE as compared to those obtained from separate analyses. In general, the estimates are comparable for HGLM and Unph for separate analyses, regardless of sample size. Using only the family data, the estimates of bias and MSE via HGLM are also comparable to CLG. We also compared the performance of our combined analysis with an existing approach based on a single marker analysis. The estimated genotype relative risk and FPR for each marker of the SCOUT analysis which is a likelihood-based approach for each single marker are listed in Table S1 (see Supplementary Material for details). The SCOUT analysis has satisfactory FPR under the null model. For a fair comparison of power with the single marker results, we further performed a global test of whether any of the haplotypes spanned by the markers are significant through HGLM, Unph and CLG. The power of the overall test under the null model is consistent to the single marker results within expectation.

### Two-SNP model with mild and moderate effects: power, BIAS and MSE

**Figure 3:**
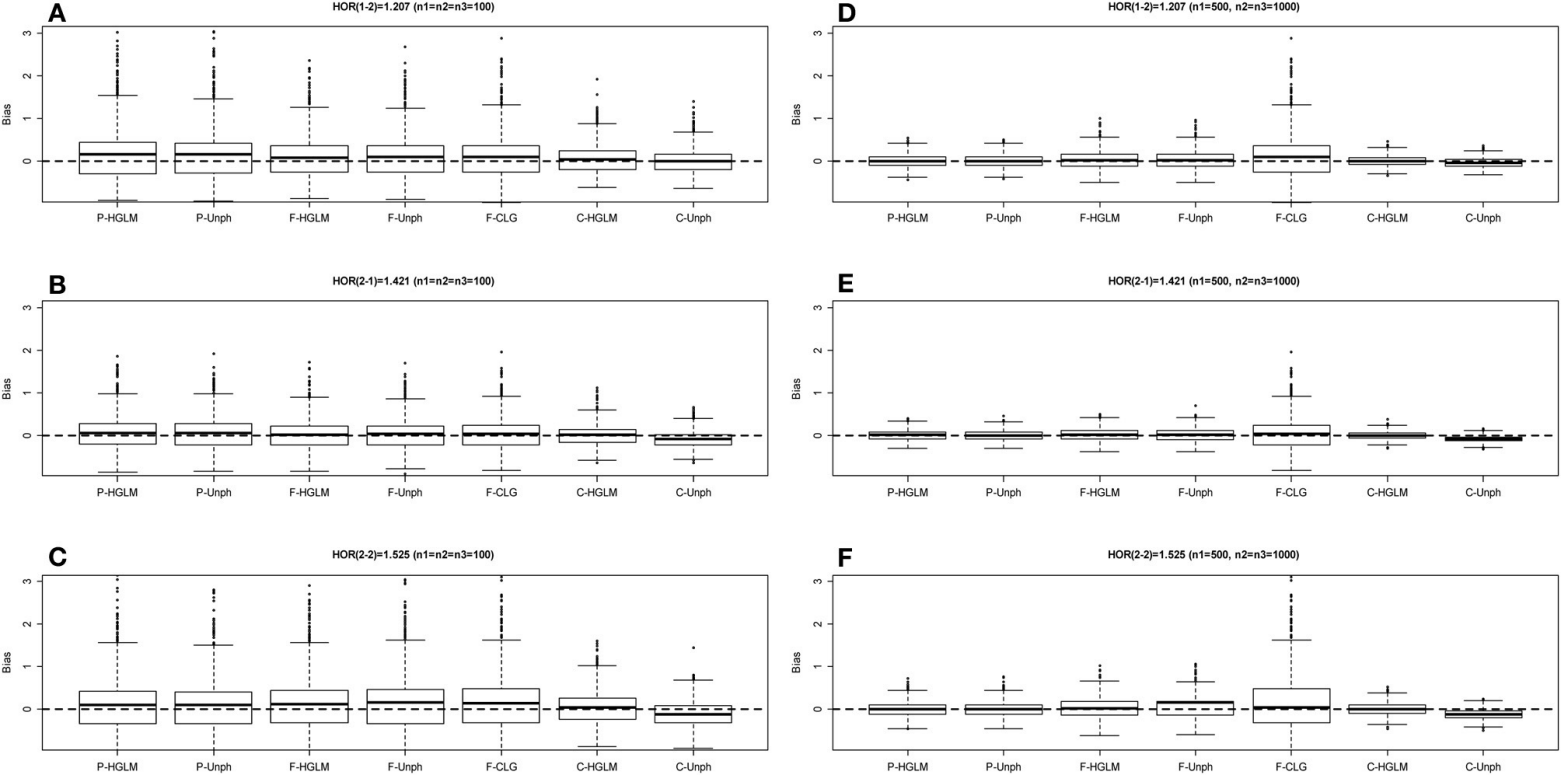
**Boxplot of estimate biases (estimate - true HOR) for haplotype odds ratios (HOR) based on mild effect model over 1000 replications**. **(A–C)** Denotes the HOR = 1.207, 1.421, 1.525 for *n*_1_ = *n*_2_ = *n*_3_ = 100 and **(D–F)** is HOR = 1.207, 1.421, 1.525 for *n*_1_ = 500, *n*_2_ = *n*_3_ = 1000.

**Figure 4:**
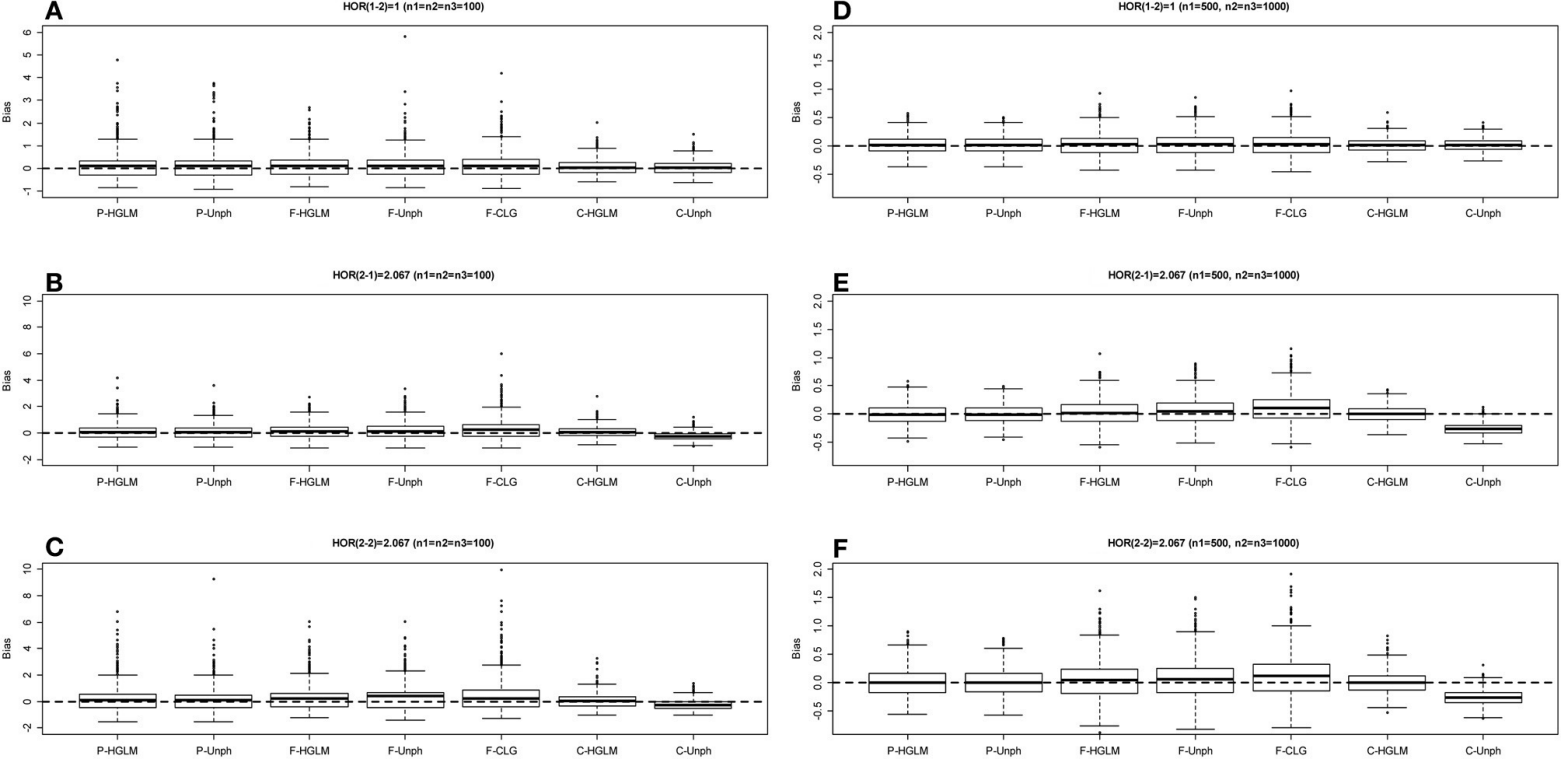
**Boxplot of estimate biases (estimate—true HOR) for haplotype odds ratios (HOR) based on moderate effect model over 1000 replications**. **(A–C)** Denotes the HOR = 1, 2.067, 2.067 for *n*_1_ = *n*_2_ = *n*_3_ = 100 and **(D–F)** is HOR = 1, 2.067, 2.067 for *n*_1_ = 500, *n*_2_ = *n*_3_ = 1000.

Table 2 provides the power for HGLM and Unph under mild and moderate effect models. Based on the combined data analysis, the power of the global haplotype test from either HGLM or Unph increases steadily with sample size, but is slightly lower than power of SNP 1 from SCOUT (details in Table S1 in the Supplementary Material). However, the power of the overall test via HGLM is comparable to that of SNP 1 adjusted by Bonferroni correction via SCOUT at small sample size (*n*_1_ = *n*_2_ = *n*_3_ = 100). It is worth mentioning that the effect of SNP 2 has been missed based on SCOUT, which was expected as a single marker analysis might not be reliable for detection of joint marker effects (i.e., haplotype effects exist). With regard to estimation, the results based on combined data research-article HGLM is as accurate and as efficient as compared to Unph (as shown in Figures 3, 4). For HGLM using combined data, the estimators of HOR have similar MSE as compared to Unph, however, the values of bias from HGLM are smaller than those from Unph when the risk is moderate. As noted in previous reports (Schaid, 2004; Xie and Stram, 2005), this is due to variations on likelihood-based methods for estimating haplotype-specific risks away from the null hypothesis. The confidence intervals based on combined data obtained from HGLM have correct coverage probabilities. For Unph, the slightly large biases contribute to slightly lower coverage probabilities (details in Table S2 in the Supplementary Material).

**Table 2:** **FPR and Power (%) at 5% significance level for (1) 2-locus mild effect model and (2) moderate effect model under varying sample size**.

**Combined study**	**Sample size**	**Haplotype**	**True HOR**	**Power (%)**	**Population data**		**Family data**			**Combined data**	
					**P-HGLM**	**P-Unph**	**F-HGLM**	**F-Unph**	**F-CLG**	**C-HGLM**	**C-Unph**
Mild	*n*_1_ = 100			Overall	25.6	26.2	27.2	27.1	27.1	50.2	38.6
Effect	*n*_2_ = *n*_3_ = 100	1–2	1.207	Haplotype	6.6	7.1	7.3	7.4	6.7	9.1	9.9
Model		2-1	1.421	Haplotype	27.3	28.1	27.4	27.8	27.6	50.8	41.5
		2-2	1.525	Haplotype	18.4	18.1	24.3	23.4	23.1	38.1	32.2
	*n*_1_ = 500			Overall	100	100	92.1	92.2	92.3	100	100
	*n*_2_ = *n*_3_ = 1000	1-2	1.207	Haplotype	27.7	27.2	22.0	21.6	22.5	42.8	38.2
		2-1	1.421	Haplotype	99.4	99.3	90.4	90.2	90.4	99.8	99.8
		2-2	1.525	Haplotype	95.3	95.4	79.4	78.4	78.4	99.5	98.9
Moderate	*n*_1_ = 100			Overall	87.4	87.6	86.4	86.0	87.0	99.3	97.7
Effect	*n*_2_ = *n*_3_ = 100	1-2	1^a^	Haplotype	3.9	3.8	5.3	4.6	4.4	5.2	4.8
Model		2-1	2.067	Haplotype	82.2	83.0	87.8	88.0	88.2	99.2	96.8
		2-2	2.067	Haplotype	46.5	47.7	54.7	54.5	53.6	84.1	75.7
	*n*_1_ = 500			Overall	100	100	100	100	100	100	100
	*n*_2_ = *n*_3_ = 1000	1-2	1^a^	Haplotype	4.1	4.2	5.4	5.5	5.4	5.5	5.6
		2-1	2.067	Haplotype	100	100	100	100	100	100	100
		2-2	2.067	Haplotype	100	100	99.8	99.8	99.8	100	100

a The power under true HOR = 1 represents the FPR.

The reference haplotype is 1-1.

### Multi-SNP model: power, BIAS and MSE

Table 3 provides bias and power for a 5-SNP haplotype-specific association analysis. The estimates via HGLM are biased upward as compared to Unph based on a small sample size when a large number of haplotypes are studied. This is due to the HFs are incorrectly estimated with small samples. The magnitude of estimation bias would be small and comparably well to Unph based on a large sample size. Given the current large sample sizes in genetic studies, HGLM will lead to less bias. Besides, the estimates from Unph for high-risk haplotypes are biased upward, similar to the results for 2-SNP model with moderate risks. For multi-SNP models, the powers of the global haplotype test and the haplotype-specific association tests from either HGLM or Unph increases with sample size (Table 3). HGLM performs comparably well to Unph in terms of power. We conclude from the results that HGLM offers a reliable approach to test for multi-SNP haplotypic associations for combining unrelated case-control and trios, except with small samples for multi-SNP haplotypes.

**Table 3:** **Power (%) at 5% significance level and bias for the 5-locus model under varying sample size. The reference haplotype is 1-2-1-2-2**.

**Sample size**	**Haplotype**	**True HOR**	**Estimate**	**Population data**		**Family data**			**Combined data**	
				**P-HGLM**	**P-Unph**	**F-HGLM**	**F-Unph**	**F-CLG**	**C-HGLM**	**C-Unph**
*n*_1_ = 100			Overall power	98.1	98.0	98.2	98.5	98.3	100	100
*n*_2_ = *n*_3_ = 100	1-1-1-1-1	1.467	Power	9.5	9.7	8.8	9.9	8.0	14.4	14.0
			Bias	0.502	0.384	0.310	0.350	0.488	0.157	0.057
	1-1-2-1-1	3.811	Power	97.2	97.7	97.0	97.9	97.5	100	99.5
			Bias	0.355	0.215	0.493	0.571	1.311	0.160	−0.904
	1-2-2-2-2	1.528	Power	17.7	19.0	20.2	19.4	17.7	33.5	30.4
			Bias	0.178	0.149	0.149	0.158	0.252	0.075	−0.031
	2-2-2-2-1	1.309	Power	9.8	10.0	11.2	10.3	10.6	16.6	14.7
			Bias	0.120	0.096	0.101	0.126	0.176	0.045	−0.003
	2-2-2-2-2	1.501	Power	14.3	14.3	12.0	12.4	12.1	22.0	20.5
			Bias	0.364	0.311	0.255	0.284	0.418	0.137	0.028
*n*_1_ = 500			Overall power	100	100	100	100	100	100	100
*n*_2_ = *n*_3_ = 1000	1-1-1-1-1	1.467	Power	47.9	49.4	29.8	32.4	30.4	68.9	68.1
			Bias	0.052	0.042	0.043	0.060	0.081	0.026	−0.032
	1-1-2-1-1	3.811	Power	100	100	100	100	100	100	100
			Bias	−0.049	−0.067	0.097	0.138	0.457	−0.031	−0.940
	1-2-2-2-2	1.528	Power	92.3	93.4	75.3	74.4	74.0	99.4	98.2
			Bias	0.005	−0.001	0.029	0.023	0.064	0.003	−0.080
	2-2-2-2-1	1.309	Power	64.4	65.5	38.6	38.1	37.6	83.3	78.2
			Bias	0.008	0.001	0.014	0.015	0.035	0.002	−0.041
	2-2-2-2-2	1.501	Power	71.7	73.8	44.4	44.9	43.6	89.2	86.9
			Bias	0.025	0.019	0.043	0.051	0.092	0.016	−0.060

## Performance in the presence of population stratification

We conducted an additional set of simulations to examine the PS effect with two sample sizes at *n*_1_ = *n*_2_ = *n*_3_ = 100, and *n*_1_ = 500 and *n*_2_ = *n*_3_ = 1000. Similar to previous studies (Epstein et al., 2005; Chen and Lin, 2008), the first set of simulations is that the population cases and controls are sampled from a mixed population composed of two ancestral populations as the first population had HFs corresponding to haplotypes (1-1, 1-2, 2-1, 2-2) at (0.5, 0.1, 0.3, 0.1) and the prevalence of disease was 7% while the second population had HFs at (0.4, 0.3, 0.15, 0.15) and the prevalence of disease was 18%. We simulated two populations with different disease and HFs with constituent proportions (50, 50%), and then randomly sampled unrelated cases and controls from the admixed population at large. Nevertheless, the trios are sampled from one population with HFs at (0.4, 0.3, 0.15, 0.15). The second scenario considers generating admixed population cases, controls and trios from the same admixed population as previous setting. There exists no haplotype-specific association (HOR = 1) under the two scenarios. The PS came from heterogeneities within components of combined data, that is, admixture of population case-control data and/or trios results in the presence of PS. We assessed the power of detecting PS by using the ANOVA framework based on the continuous haplotype weighted-count data. For Unph, a sample indicator variable was included (confounder option) as a covariate to test and account for PS, and the power could be calculated from the “offset column” which models a change in HFs compared to that at the baseline covariate level. Table 4 shows the power of the tests for checking the appropriateness of combining samples when PS exists. In the first scenario considered, the proposed test has good power for detecting the inappropriateness of combining two different samples while the powers of ANOVA and Unph decrease in the second scenario. As expected, the powers under the proposed ANOVA and Unph depend on the amounts of discrepancy among HFs. Furthermore, when sample size increases, the proposed ANOVA and Unph lead to larger power. When there is PS, the overall test (i.e., MANOVA) and haplotype-specific test (i.e., ANOVA) for appropriateness of combined data perform better than Unph in terms of larger power.

**Table 4:** **Power of the tests for checking the appropriateness of combining samples in presence of population stratification (PS)**.

**Sample size**	**Haplotype**	**True HOR**	**Power (%)**	**PS^**1**^**		**PS^**2**^**	
				**ANOVA**	**Unph (Confounder)**	**ANOVA**	**Unph (Confounder)**
*n*_1_ = 100			overall	0.958^a^	0.705	0.130^a^	0.058
*n*_2_ = *n*_3_ = 100	1-2	1	haplotype	0.865	0.595	0.082	0.057
	2-1	1	haplotype	0.842	0.250	0.087	0.051
	2-2	1	haplotype	0.325	0.257	0.105	0.043
*n*_1_ = 500			overall	1^a^	1	0.208^a^	0.086
*n*_2_ = *n*_3_ = 1000	1-2	1	haplotype	1	1	0.097	0.067
	2-1	1	haplotype	1	0.956	0.102	0.072
	2-2	1	haplotype	0.932	0.939	0.157	0.059

PS^1^: Population cases and controls are randomly ascertained from admixed population consisting of two strata with disease prevalence (7, 18%) and HFs for Haplotype 1-1, 1-2, 2-1, and 2-2 at (0.5, 0.1, 0.3, 0.1) and (0.4, 0.3, 0.15, 0.15), respectively. Trios are sampled from one ancestral population with HFs at (0.4, 0.3, 0.15, 0.15). PS^2^: Population cases, controls and trios are randomly ascertained from the same admixed population as PS^1^:

a Represents the power of MANOVA.

We then examined the FPRs for the modified HGLM (M-HGLM) with clustering techniques in the two scenarios with PS. Tables 5, 6 display the FPRs for HGLM based on only family trio data (F-HGLM), and HGLM without modification (C-HGLM), M-HGLM with Kmeans [M-HGLM (Kmeans)] and Ward approach [M-HGLM (Ward)], and Unphased with confounder option [C-Unph (confounder)] based on admixed combined data for two sample sizes at *n*_1_ = *n*_2_ = *n*_3_ = 100, and *n*_1_ = 500 and *n*_2_ = *n*_3_ = 1000, respectively. The result reveals that F-HGLM using only family trio data is free of the effect of PS in terms of the bias and FPR under the two scenarios as expected. On the contrary, we can see that the FPRs from C-HGLM are much greater than the nominal level, 0.05, in the presence of PS. Incorporating clustering techniques with HGLM, M-HGLM (Kmeans) and M-HGLM (Ward) both perform similar to C-Unph (confounder) in terms of adequate FPR and small bias even when sample size is small. The result indicates that HGLM modified by clustering methods in advance leads to valid inference in the presence of PS. Moreover, we can see from Tables 4–6 that M-HGLM can control for PS even when weak evidence for PS is obtained (e.g., the second set of simulations). In other words, M-HGLM is recommended to ease the concern of PS in practice. Furthermore, we also simulated two populations with different disease and HFs as the pervious setting with constituent proportions (70, 30%), and sampled different numbers of samples for population cases and controls from the two populations. Table S3 displays the FPRs of different approaches for a large sample size at *n*_1_ = 500 and *n*_2_ = *n*_3_ = 1000 (see Supplementary Material for details). The result indicates that M-HGLM is a reliable approach in the presence of PS even when the numbers of samples for population cases and controls from the two populations are different.

**Table 5:** **False positive rates of the modified HGLM (M-HGLM) with clustering methods (Kmeans and Ward) in the presence of population stratification (*n*_1_ = *n*_2_ = *n*_3_ = 100)**.

**Scenarios**	**Haplotype**	**HOR**	**Estimate**	**F-HGLM**	**C-HGLM**	**M-HGLM (Kmeans)**	**M-HGLM (Ward)**	**C-Unph (Confounder)**
	FPR of the overall test			0.062	0.916	0.047	0.047	0.050
PS^1^	1-2	1	FPR	0.044	0.659	0.041	0.049	0.051
			Bias	0.041	−0.352	0.009	0.010	0.012
	2-1	1	FPR	0.046	0.270	0.045	0.037	0.045
			Bias	0.074	0.394	0.023	0.028	0.024
	2-2	1	FPR	0.057	0.243	0.045	0.036	0.041
			Bias	0.068	−0.238	0.028	0.023	0.025
	FPR of the overall test			0.042	0.913	0.053	0.042	0.067
PS^2^	1-2	1	FPR	0.039	0.781	0.055	0.042	0.055
			Bias	0.031	−0.415	0.004	0.006	0.021
	2-1	1	FPR	0.047	0.178	0.049	0.049	0.045
			Bias	0.029	0.244	0.008	0.010	0.018
	2-2	1	FPR	0.040	0.192	0.050	0.046	0.052
			Bias	0.063	−0.225	0.027	0.021	0.041

PS^1^: Population cases and controls are randomly ascertained from admixed population consisting of two strata with disease prevalence (7%, 18%) and HFs for Haplotype 1-1, 1-2, 2-1, and 2-2 at (0.5, 0.1, 0.3, 0.1) and (0.4, 0.3, 0.15, 0.15), respectively. Trios are sampled from one ancestral population with HFs at (0.4, 0.3, 0.15, 0.15). PS^2^: Population cases, controls and trios are randomly ascertained from the same admixed population as PS^1^.

**Table 6:** **False positive rates of the modified HGLM (M-HGLM) with clustering methods (Kmeans and Ward) in the presence of population stratification (*n*_1_ = 500 and *n*_2_ = *n*_3_ = 1000)**.

**Scenarios**	**Haplotype**	**HOR**	**Estimate**	**F-HGLM**	**C-HGLM**	**M-HGLM (Kmeans)**	**M-HGLM (Ward)**	**C-Unph (Confounder)**
	FPR of the overall test			0.037	0.590	0.050	0.050	0.057
PS^1^	1-2	1	FPR	0.054	0.390	0.048	0.037	0.056
			Bias	0.009	−0.112	0.001	0.002	0.002
	2-1	1	FPR	0.051	0.146	0.055	0.046	0.050
			Bias	0.013	0.073	0.001	0.003	0.003
	2-2	1	FPR	0.053	0.133	0.043	0.055	0.058
			Bias	0.015	−0.069	0.006	0.004	0.004
	FPR of the overall test			0.037	0.612	0.035	0.041	0.049
PS^2^	1-2	1	FPR	0.030	0.457	0.046	0.038	0.048
			Bias	0.008	−0.127	0.001	0.002	0.002
	2-1	1	FPR	0.044	0.105	0.038	0.048	0.043
			Bias	0.006	0.057	<0.001	0.002	0.002
	2-2	1	FPR	0.052	0.117	0.052	0.052	0.061
			Bias	0.014	−0.067	0.006	0.003	0.004

PS^1^: Population cases and controls are randomly ascertained from admixed population consisting of two strata with disease prevalence (7%, 18%) and HFs for Haplotype 1-1, 1-2, 2-1, and 2-2 at (0.5, 0.1, 0.3, 0.1) and (0.4, 0.3, 0.15, 0.15), respectively. Trios are sampled from one ancestral population with HFs at (0.4, 0.3, 0.15, 0.15). PS^2^: Population cases, controls and trios are randomly ascertained from the same admixed population as PS^1^.

## Discussion

Nowadays the combination of available data including both trios and population case-control subjects is facilitated from existing nationwide registries of families and additional validation dataset was commonly used in a genetic association study. Most of previous methods were developed for combined test of association based on a single marker test. However, an analysis of haplotypes that combined information from multiple markers could be more powerful than a single-marker test. For this reason, we have developed the HGLM approach for combining data from both family trios and unrelated case-controls to increase power to detect the haplotype specific association on disease. To examine the haplotype effect, we adopt the haplotype weighted-counts as covariates. The strength of haplotype weighted-counts is to assess the suitability of combined family trios and population case-control samples naturally by comparing the equality of HFs for each data source. HGLM is a simpler model after adjusting for haplotype covariates, and easier to estimate and test for haplotype-specific effects, as well as easily done with conventional software once haplotype weighted-count data are obtained. Particularly, the modified HGLM by clustering methods on mixed case-control samples could further take into account of the bias of PS.

Our simulation results show that HGLM performs nearly as efficient as the Unphased implementation in estimation and test of the disease risk from combined data under the null model. Furthermore, the bias, coverage rate and power of HGLM are the same as or slightly better than those of the Unph implementation keeping the sample size fixed, particularly when the disease risk exists. Unlike previous likelihood-approaches based on the rare disease assumption, our proposed approach requires few assumptions. In simulations, we found that HGLM would be more appropriate than SCOUT to detect association with the joint-marker effect for a rare disease in a homogeneous population. The proposed HGLM approach would be a suitable strategy under conditions that most risk loci for complex traits appear to have small to very small effects or rare genetic effects that are grouped together on haplotypes. It is worth noting that the proposed approach is comparable to CLG when using only family data.

When there is PS in the data, formally examination of PS may be done through an analysis of variance framework based on haplotype weighted-counts. This is equivalent to check the equality of the HFs estimated from population case-control data and family trios separately; thus, it might serve as an alternative approach for testing whether samples can be combined safely. The simulation results imply that the proposed test performs better than Unphased in the presence of PS. Utilizing continuity of haplotype weighted-counts, the power gain on the basis of ANOVA testing is substantial. To account for PS, we further recommend clustering mixed case-control samples in advance, and to estimate haplotype weighted-count data from homogeneous subgroups. Strength of clustering methods is that it makes use of existing markers that are known to differ in frequencies between mixed case-control samples, and thereby the modified HGLM could be robust against PS. As observed in the previous study (Chung et al., 2010), the performance of clustering techniques remains robust even when the number of clusters is specified as more than the true number of subpopulations. Generally, however, the number of subpopulations is unknown. For hierarchical clustering methods (e.g., Ward's method), one can determine the number of clusters based on a number of statistics such as R-square, semipartial R-square, and distance between two clusters. For non-hierarchical clustering methods (e.g., K-mean algorithm), it is recommended that one can use an *a priori* knowledge of the number of clusters from the results of hierarchical methods or of pervious researches to further refine the cluster solution. Another consideration is the accuracy of clustering of subpopulations. In our simulation studies, the correct clustering rates for Ward clustering and K-mean algorithm are not high (the clustering error rates of Ward clustering and K-mean algorithm are nearly 40 and 47%, respectively). Not surprisingly, it results from that we only used 2 SNPs to infer population structure. However, the clustering technique does substantially eliminate the bias arising from PS. As the number of markers increases, the accuracy of clustering is expected to be improved.

With current technology it is difficult to determine the phase of genotype data, this has led to a variety of statistical approaches inferring the probabilities of the possible haplotypes. One issue would be the accuracy of the estimation of haplotypes from the genotype data, especially for the population case-control data. Although the efficiency of HGLM will depend on which methods for inferring the haplotype weight, the proposed method has negligible loss of power compared with that based on known haplotype (data not shown). Another issue is related to rare haplotypes for haplotype-based association studies. As for extending to more loci in recent studies, lengthy haplotypes may lead to rare haplotypes. We suggest either to discard the rare haplotypes or to combine all rare ones for a further analysis. However, the interpretation of the regression coefficients might be different according to the elements of a specific reference haplotype. Our methods are developed and evaluated for binary traits, but they can be easily extended to quantitative traits in the framework of generalized linear models. Besides, the proposed HGLM method may be extended further for addressing realistic scenarios such as including genetic-environment interactions and complex pedigrees. For the purpose of involving genetic-environment interactions in HGLM, great care must be taken to define the environmental effects for family trios and to consider the mode of inheritance of haplotype effects. As an extension to handle complex pedigrees, HGLM can be modified as a generalized linear mixed model by specifying the covariance structure among the members of a pedigree. Another consideration is to deal with incomplete genotype data, such as incomplete trios which parental data are usually not available for late-onset diseases. Currently, strategies for imputing genotype data are proposed such as a unified framework that enables the simultaneous use of data from trios and unrelated individuals (Browning and Browning, 2009). Thus, HGLM can be implemented on the basis of genotype imputation. Additional research focusing on these aspects would be needed and there remains much work to be done to meet these complicating issues and challenges.

### Conflict of interest statement

The authors declare that the research was conducted in the absence of any commercial or financial relationships that could be construed as a potential conflict of interest.
