# Supplementary material for: Haplotype association analysis of combining unrelated case-control and triads with consideration of population stratification
Source: Front Genet. 2014 Apr 29;5:103. doi: 10.3389/fgene.2014.00103 (PMC4028876; doi:10.3389/fgene.2014.00103)
Supplement: Supplementary file 1 [file Presentation1.PDF]

Supplementary Material

TABLE S1: Estimated mean genotype relative risk (GRR) and power of the combined association test in Epstein et al. [10] under the 2-locus model over 1000 replications. The haplotype ORs are 1.207, 1.421 and 1.525 in the mild effect model, and 1, 2.067 and 2.067 in the moderate effect model. Null model represents the haplotype ORs equal to 1. The reference haplotype is 1-1.

			Additive model			Multiplicative model		
Combined study	Sample size	Marker	GRR	Power	Power[B]	GRR	Power	Power[B]
null model[a]	$n_1 = 100$	SNP 1	1.022	0.060	0.031	1.013	0.063	0.034
	$n_2 = n_3 = 100$	SNP 2	1.019	0.058	0.033	1.013	0.064	0.032
	$n_1 = 500$	SNP 1	1.000	0.052	0.034	0.999	0.051	0.032
	$n_2 = n_3 = 1000$	SNP 2	1.000	0.043	0.022	1.000	0.043	0.023
mild effect model	$n_1 = 100$	SNP 1	1.470	0.623	0.511	1.398	0.621	0.519
	$n_2 = n_3 = 100$	SNP 2	1.213	0.184	0.112	1.201	0.186	0.111
	$n_1 = 500$	SNP 1	1.455	0.992	0.992	1.400	0.998	0.998
	$n_2 = n_3 = 1000$	SNP 2	1.190	0.731	0.638	1.184	0.732	0.637
moderate effect model	$n_1 = 100$	SNP 1	2.475	1.000	0.995	2.076	0.999	0.995
	$n_2 = n_3 = 100$	SNP 2	1.101	0.068	0.042	1.093	0.070	0.044
	$n_1 = 500$	SNP 1	2.396	1	1	2.057	1	1
	$n_2 = n_3 = 1000$	SNP 2	1.099	0.286	0.194	1.096	0.286	0.190
5snp	$n_1 = 100$	SNP 1	0.721	0.751	0.513	0.672	0.756	0.522
	$n_2 = n_3 = 100$	SNP 2	0.612	1.000	0.999	0.411	1.000	0.999
		SNP 3	2.506	0.895	0.719	1.845	0.890	0.724
		SNP 4	0.612	1.000	0.999	0.411	1.000	0.999
		SNP 5	0.687	0.943	0.835	0.605	0.947	0.846
	$n_1 = 500$	SNP 1	0.712	1.000	1.000	0.665	1.000	1.000
	$n_2 = n_3 = 1000$	SNP 2	0.609	1.000	1.000	0.407	1.000	1.000
		SNP 3	2.483	1.000	1.000	1.798	1.000	1.000
		SNP 4	0.609	1.000	1.000	0.407	1.000	1.000
		SNP 5	0.681	1.000	1.000	0.597	1.000	1.000

[a]: the power under null model represents the FPR. Power[B] is estimated based on the Bonferroni correction for multiple tests.

TABLE S2: Estimates of haplotype odds ratios (HOR) obtained from the proposed HGLM approach based on (1) 2-locus mild effect model and (2) moderate effect model under varying sample size. The reference haplotype is 1-1. Bias and MSE are bias and mean square error of estimates for haplotype odds ratio. CR denotes the coverage rate of 95% confidence interval for haplotype odds ratio.

					Population Data		Family Data			Combined Data	
Combined study	Sample size	Haplotype	True HOR	Estimate	P-HGLM	P-Unph	F-HGLM	F-Unph	F-CLG	C-HGLM	C-Unph
mild effect model	$n_1 = 100$ $n_2 = n_3 = 100$	1-2	1.207	Bias	0.155	0.150	0.086	0.103	0.108	0.047	-0.004
				MSE	0.471	0.448	0.252	0.279	0.296	0.124	0.089
				CR	0.958	0.952	0.957	0.953	0.956	0.965	0.964
		2-1	1.421	Bias	0.058	0.053	0.021	0.029	0.043	0.005	-0.092
				MSE	0.158	0.151	0.126	0.135	0.150	0.057	0.045
				CR	0.954	0.950	0.956	0.947	0.956	0.958	0.936
		2-2	1.525	Bias	0.108	0.100	0.125	0.137	0.163	0.044	-0.113
				MSE	0.464	0.452	0.436	0.481	0.562	0.157	0.095
				CR	0.952	0.959	0.944	0.943	0.946	0.959	0.999
	$n_1 = 500$ $n_2 = n_3 = 1000$	1-2	1.207	Bias	0.002	0.001	0.024	0.022	0.032	0.003	-0.036
				MSE	0.025	0.024	0.044	0.045	0.049	0.015	0.012
				CR	0.955	0.953	0.948	0.945	0.944	0.957	0.946
		2-1	1.421	Bias	0.005	0.003	0.007	0.009	0.019	0.002	-0.087
				MSE	0.013	0.013	0.023	0.024	0.026	0.008	0.013
				CR	0.953	0.952	0.945	0.947	0.949	0.960	0.799

		2-2	1.525	Bias	0.009	0.007	0.030	0.036	0.043	0.007	-0.112
				MSE	0.032	0.031	0.060	0.064	0.066	0.021	0.026
				CR	0.958	0.961	0.959	0.956	0.960	0.952	1.000
moderate effect model	$n_1 = 100$ $n_2 = n_3 = 100$	1-2	1	Bias	0.091	0.089	0.101	0.105	0.117	0.037	0.027
				MSE	0.381	0.354	0.293	0.289	0.348	0.114	0.089
				CR	0.961	0.962	0.947	0.954	0.956	0.948	0.952
		2-1	2.067	Bias	0.079	0.061	0.132	0.150	0.278	0.050	-0.267
				MSE	0.350	0.306	0.346	0.375	0.610	0.152	0.145
				CR	0.948	0.944	0.942	0.944	0.945	0.956	0.835
		2-2	2.067	Bias	0.121	0.094	0.209	0.220	0.392	0.041	-0.274
				MSE	0.887	0.968	0.887	0.945	1.539	0.294	0.208
				CR	0.950	0.955	0.960	0.952	0.956	0.958	0.905
	$n_1 = 500$ $n_2 = n_3 = 1000$	1-2	1	Bias	0.009	0.009	0.019	0.025	0.024	0.006	0.008
				MSE	0.024	0.022	0.038	0.053	0.043	0.014	0.012
				CR	0.959	0.958	0.946	0.958	0.946	0.945	0.944
		2-1	2.067	Bias	-0.002	-0.005	0.024	0.050	0.105	<0.001	-0.260
				MSE	0.029	0.027	0.051	0.053	0.078	0.018	0.077
				CR	0.944	0.941	0.958	0.958	0.936	0.949	0.905
		2-2	2.067	Bias	0.003	<0.001	0.043	0.055	0.121	0.003	-0.263
				MSE	0.058	0.056	0.111	0.113	0.158	0.037	0.087
				CR	0.956	0.955	0.956	0.960	0.949	0.954	1.000

TABLE S3 False positive rates of the modified HGLM (M-HGLM) with clustering methods (Kmeans and Ward) in the presence of population stratification ($n_1 = 500$ and $n_2 = n_3 = 1000$) when the numbers of samples for population cases and controls from the two populations are different.

Scenarios	Haplotype	HOR	Estimate	F-HGLM	C-HGLM	M-HGLM (Kmeans)	M-HGLM (Ward)	C-Unph (Confounder)
	FPR of the overall test			0.037	1	0.039	0.045	0.0.61
PS[3]	1-2	1	FPR	0.054	1	0.051	0.036	0.059
			Bias	0.009	0.561	0.003	0.003	0.031
	2-1	1	FPR	0.051	0.677	0.045	0.046	0.055
			Bias	0.013	-0.163	0.004	0.004	0.028
	2-2	1	FPR	0.053	0.789	0.052	0.048	0.047
			Bias	0.015	0.280	0.005	0.003	0.014

PS[3]: We simulated two populations with different disease and haplotype frequencies with constituent proportions (70%, 30%). The numbers of cases and controls are (700, 700) from the first population with disease prevalence at 7% and HFs for Haplotype 1-1, 1-2, 2-1, and 2-2 at (0.5, 0.1, 0.3, 0.1). A total of 300 cases and 300 controls generated from the second population with disease prevalence at 18% and HFs for Haplotype 1-1, 1-2, 2-1, and 2-2 at (0.4, 0.3, 0.15, 0.15). Trios are sampled from one ancestral population with HFs at (0.4, 0.3, 0.15, 0.15).
